# Analysis of Knee Joint Injury Caused by Physical Training of Freshmen Students Based on 3T MRI and Automatic Cartilage Segmentation Technology: A Prospective Study

**DOI:** 10.3389/fendo.2022.839112

**Published:** 2022-05-09

**Authors:** Lingling Liu, Henan Liu, Zhiming Zhen, Yalan Zheng, Xiaoyue Zhou, Esther Raithel, Jiang Du, Yan Hu, Wei Chen, Xiaofei Hu

**Affiliations:** ^1^ Department of Radiology, First Affiliated Hospital of Army Medical University, Chongqing, China; ^2^ Department of Nuclear Medicine, First Affiliated Hospital of Army Medical University, Chongqing, China; ^3^ MR Collaboration, Siemens Healthineers Ltd., Shanghai, China; ^4^ MR Application Predevelopment, Siemens Healthcare GmbH, Erlangen, Germany; ^5^ Health Service Training Base, Army Medical University, Chongqing, China

**Keywords:** knee joint injury, automatic cartilage segmentation technology, three-dimensional quantitative MRI, cartilage, athletic injury

## Abstract

**Background:**

The differential effects of various exercises on knee joint injury have not been well documented. Improper physical training can cause irreversible damage to the knee joint. MRI is generally used to precisely analyze morphological and biochemical changes in the knee cartilage. We compared the effects of long-walking and regular daily physical training on acute and chronic knee joint injuries as well as cartilage structure in freshmen students.

**Methods:**

A total of 23 young male college freshmen were recruited to participate in an 8-day 240 km long distance walk and a one-year daily training. 3D-DESSwe, 2D T_2_ mapping, DIXON, and T1WI of the right knee joint were performed using the MAGNETOM Spectra 3T MR scanner. The injury of meniscus, bone marrow edema, ligaments and joint effusion is graded. Cartilage volume, thickness and T2 values of 21 sub-regions of the knee cartilage were estimated using automatic cartilage segmentation prototype software. Friedman’s test and Wilcoxon paired rank-sum test were used to compare quantitative indices of knee cartilage in three groups.

**Results:**

The injury to the medial meniscus and anterior cruciate ligament of the knee joint, joint effusion, and bone marrow edema was significantly higher in the long-walking group compared to the baseline and daily groups. Furthermore, injury to the lateral meniscus was significantly worse in the long-walking group compared to the baseline group but was significantly better in the daily group compared to the baseline group. No significant changes to the posterior cruciate ligament were observed among the three groups. Knee cartilage volume was significantly increased, mainly in the stress surface of the femur, patella, and the lateral area of the tibial plateau. Regular daily training did not significantly change the thickness of the knee cartilage. Conversely, knee cartilage thickness decreased in the long-walking group, especially in the medial and lateral areas of the femur and tibial plateau. Moreover, no significant changes were observed in the knee cartilage volume of the long-walking group. Both long-walking and daily groups showed reduced T2 values of the knee joint compared to the baseline.

**Conclusion:**

Among freshmen students and the training of this experimental intensity, our results show that regular daily training does not cause high-level injury to the knee joint, but improve the knee joint function adaptability by increasing cartilage volume. Moreover, knee injury caused by short-term long walking can be reversible.

## Introduction

According to China’s “Outline of the National Fitness Program” and the positive Olympic spirit, with increasing public health awareness, fitness is primarily started from children and adolescents and is aimed at improving overall health. It has been recommended that people should participate in more than one physical fitness activity per day, learn more than two fitness methods, and conduct a physical fitness test once per year. At present, it is common for colleges and universities to carry out freshman training in China. Knee joint injuries are common in physical training, and some injuries are irreversible (1). Different forms of motion can cause different knee joint injuries due to different knee joint mechanics (2, 3). Studies have found that the incidence of knee joint pain is significantly higher in people who regularly train on long-distance walking (4), and accumulated high-intensity walking training can directly lead to meniscus injury (5).

Magnetic resonance imaging (MRI) is the most effective technique to identify articular cartilage injury, meniscus lesion, ligament injury, bone marrow edema, and joint effusion (6–9). Three-dimensional quantitative MRI (3D q-MRI) technology, such as 3D fast imaging with steady-state precession (FISP), 3D double-echo steady-state precession (3D-DESS), 3D SPACE sequence, can precisely analyze cartilage morphology. Cartilage volume and thickness and T2 values can be measured in a high-precision and repeatable manner (8, 10–12). Previous studies of our research group also used automatic cartilage segmentation technology to obtain good repeatable automatic cartilage segmentation results using high-spatial-resolution 3.0T MR imaging (13). During the software cartilage segmentation process, we need one morphological dataset (3D-DESSwe) and optionally biochemical maps for evaluation (T_2_ mapping). Three-dimensional double-echo steady-state (3D-DESS) includes the acquisition of two different echoes in each repetition time. The first echo is the free induction attenuation gradient-echo of fast imaging with steady-state precession (FISP). The second echo is the RF echo of reversed fast imaging with steady-state free precession (PSIF). 3D-DESS with water excitation (3D-DESSwe) combines water excitation technology with a steady-state free motion-imaging sequence and is generally used for articular cartilage and cardiovascular imaging. This sequence has the characteristics of T1 and T2 double weighting and combines several fat suppression techniques (14). There are few reports on the impact of physical training on the knee joint in college freshmen students and people with similar physical status. Herein, we compared the effects of long-walking and regular daily physical training on acute and chronic knee joint injuries as well as cartilage structure by analyzing 3D-DESSwe and T_2_ mapping images using automatic cartilage segmentation prototype software.

## Materials and Methods

### Subjects

This study was approved by the ethics committee of First Affiliated Hospital of Army Medical University (No. KY2021016), and all participants signed informed consent. Selection criteria included: (1) male undergraduates enrolled in the current year; (2) body mass index (BMI) <28 kg/m^2^; (3) no history of knee pain, trauma, and surgery; and (4) complete the training plan. Exclusion criteria included: (1) previous history of knee trauma, surgery, and infection; (2) knee pain or other positive symptoms; (3) presence of metal foreign bodies or prostheses (cardiac pacemaker, insulin pump), claustrophobia, and other contraindications of magnetic resonance examination.

Twenty-nine college freshmen students were recruited from October 2019 to October 2021. Six students were lost to follow-up, and thus, 23 students were included in the final analysis. All participants were males, aged 17-20 years old (19.48 ± 0.14), with a BMI of 22.55 ± 0.34 kg/m^2^. All students participated in the entire long-distance walking training (8 days, 240 km; altitude of 168-400 m) and regular daily training (one-year unified daily training course arranged in the school, including 2 h of swimming, 2 h of endurance, 2 h of strength, and 2 h of explosive training per week).

**Figure 1:**
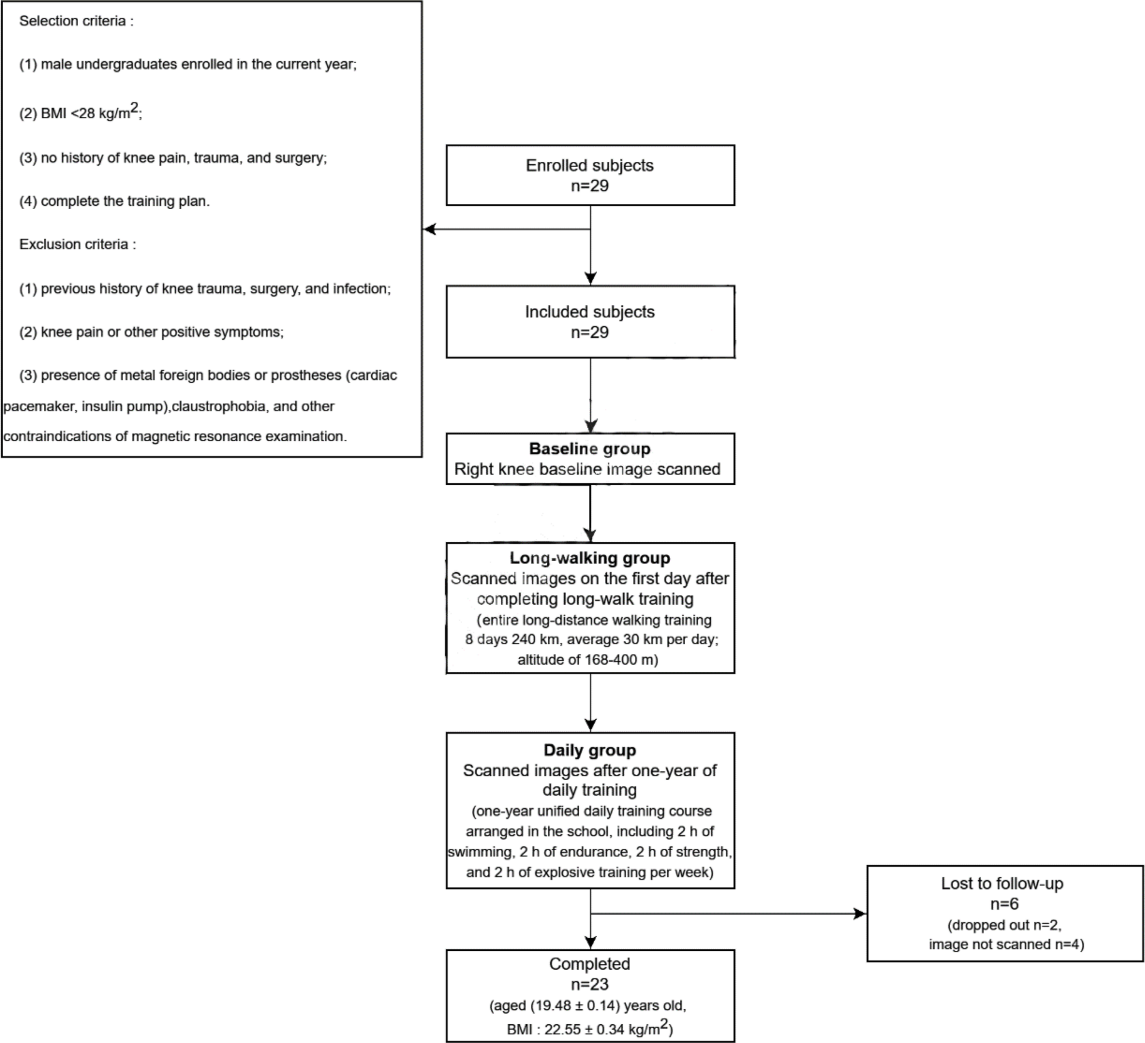
Flow chart of the study.

Participants were divided into three groups based on the three stages they experienced: before training (baseline group); one day after long-walking (long-walking group), and one year after daily physical training (daily group) (**Figure 1**).

### MR Imaging Procedures

Each participant was examined by MRI using the same technology and sequence. 3D-DESSwe, 2D T2 mapping, DIXON, and T1 weighted image (T1WI) of the right knee joint were performed using the MAGNETOM Spectra 3T MR scanner (Siemens Healthcare, Erlangen, Germany). A special 18-channel coil for the knee joint was selected. Scanning parameters were: (1) sagittal 3D-DESSwe: TR = 14.8 ms, TE = 5.3 ms, FA = 25°, slice thickness = 0.63 mm, slice spacing = 0.12 mm, FOV = 160 × 160 mm, matrix = 256 × 256, and bandwidth = 250 Hz/Px; (2) axial T2_DESSwe: TR = 15ms, TE = 5 ms, FA = 25°, slice thickness = 0.63 ms, FOV = 160 × 160 mm, matrix = 256 × 256 and bandwidth = 227 Hz/Px; (3) sagittal 2D_T_2_ _mapping: TR = 2520 ms, TE = 13.8/27.6/41.4/55.2/69.0 ms, FA = 180°, slice thickness = 2.5 mm, FOV = 160 × 160 mm, matrix = 256 × 256, and bandwidth = 227 Hz/Px; (4) sagittal T2_tse_fs-dixon: TR = 4880 ms, TE = 88 ms, FA = 138°, slice thickness = 3.5 ms, FOV = 250 × 250 mm, matrix = 256 × 256 and bandwidth = 349 Hz/Px; (5) sagittal T1WI_tse: TR = 869ms, TE = 13ms, FA = 174°, slice thickness = 2.5 ms, FOV = 160 × 160 mm, matrix = 307 × 307 and bandwidth = 150 Hz/Px. All subjects rested for one hour before each scan and underwent an examination in the supine position. The lower edge of the patella was considered as the scanning center, and sandbags and sponges were fixed to minimize motion artifacts.

### Automatic Cartilage Segmentation

**Figure 2:**
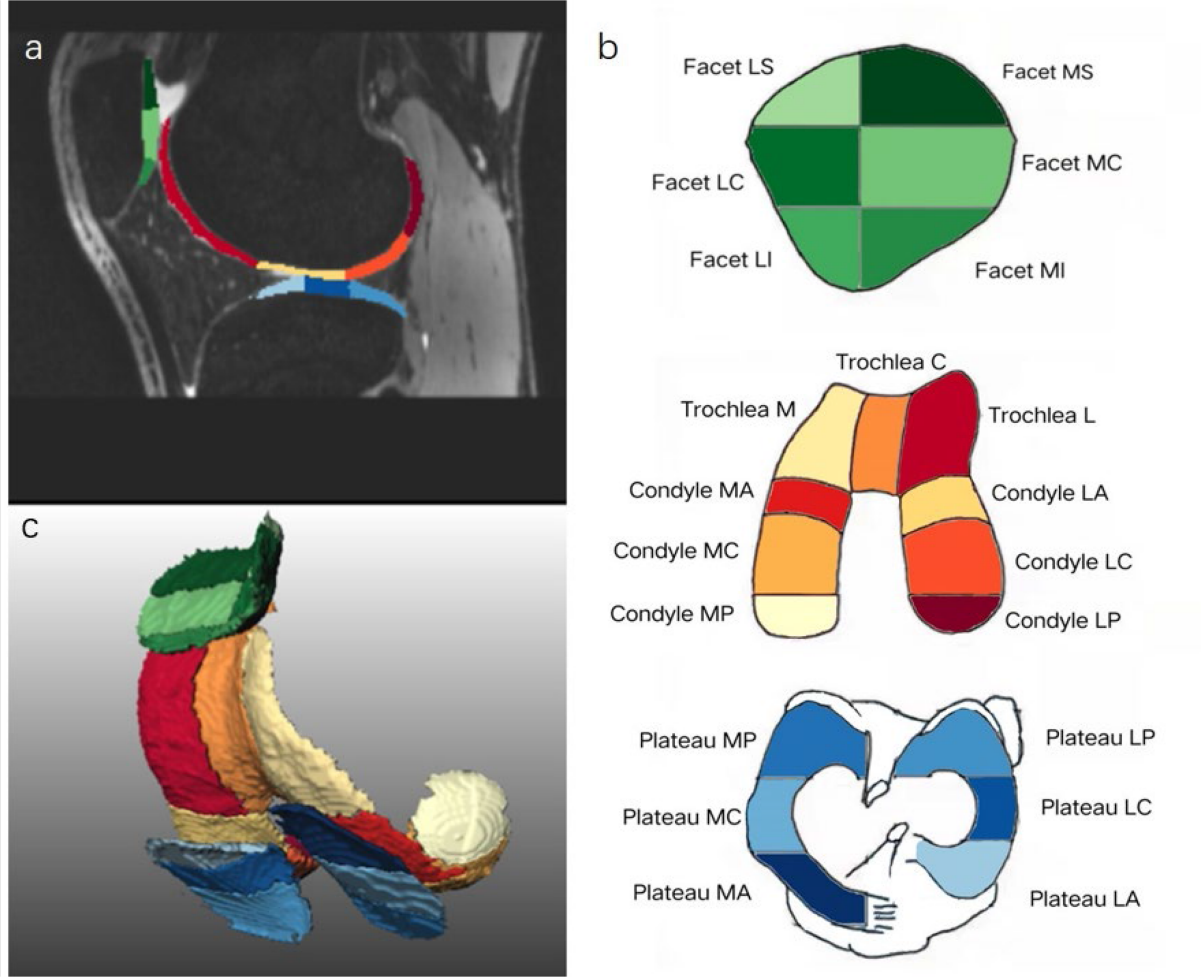
Automatic cartilage segmentation of the knee 21 cartilage sub-regions on sagittal 3D-DESSwe **(A)**, schematic diagram with colors **(B)** and 3D reconstruction image **(C)**. Green (dark green, light green) represents patellar cartilage; warm colors (red, orange, etc.) represent femoral cartilage; cold colors (light blue, dark blue, etc.) represent tibial cartilage.

Automatic cartilage segmentation prototype software (MR Chondral Health version 2.1, Siemens Healthcare, Erlangen, Germany) was used to automatically estimate cartilage volume and thickness and T2 values of 21 sub-regions of the knee cartilage from the 3D-DESSWE and 2D T_2_ mapping images. Thickness and T2 values are set as average values for each subregion. The International Cartilage Repair Society (ICRS) divides the cartilage into 21 sub-regions (15), focusing on the depth and volume of the injury (**Table 1**). The cartilage segmentation algorithm is based on the process proposed by Fripp (16). The results of cartilage volume and thickness in each sub-region were segmented (17) (**Figure 2**). After the segmentation, we performed manual and consistency checks. If the analysis results are not satisfactory, the segmentation will be repeated.

**Table 1:** Cartilage sub-regions of the knee.

	Femoral cartilage	Patellar cartilage	Tibia cartilage
Subregions	Femur condyle medial posterior (Condyle MP)	Patella facet lateral inferior (Facet LI)	Tibia plateau lateral posterior (Plateau LP)
	Femur condyle medial central (Condyle MC)	Patella facet lateral central (Facet LC)	Tibia plateau lateral central (Plateau LC)
	Femur condyle medial anterior (Condyle MA)	Patella facet lateral superior (Facet LS)	Tibia plateau lateral anterior (Plateau LA)
	Femur trochlea medial (Trochlea M)	Patella facet medial inferior (Facet MI)	Tibia plateau medial posterior (Plateau MP)
	Femur trochlea central (Trochlea C)	Patella facet medial central (Facet MC)	Tibia plateau medial central (Plateau MC)
	Femur trochlea lateral (Trochlea L)	Patella facet medial superior (Facet MS)	Tibia plateau medial anterior (Plateau MA)
	Femur condyle lateral posterior (Condyle LP)		
	Femur condyle lateral central (Condyle LC)		
	Femur condyle lateral anterior (Condyle LA)		

### Image Analysis

MR images, including meniscus, cartilage, bone marrow edema and ligaments (including anterior and posterior cruciate ligament), and joint effusion, were analyzed by two experienced musculoskeletal radiologists specialized in MRI (with over 5 and 10 years of experience). In case of discrepancies, a joint analysis was performed and a consensus decision was reached. Meniscus and cartilage damage, bone marrow edema, ligament injury, and joint effusion were graded.

Meniscus injury was scored on a 5-point scale ranging from 0 to 4. Level 0 is normal. Level 1 lesions are punctate hyperintensities that are not connected to the articular surface. Level 2 lesions are defined as linear hyperintensities that do not reach the articular surface. Level 3 lesions are linear hyperintensities that extend to one articular surface of the meniscus (meniscus tear). Level 4 lesions refer to signal intensities that reach the upper and lower surfaces of one meniscus (complex meniscus tear) (18).

According to the Outerbridge classification system, cartilage damage on MR images was scored on a 5-point scale ranging from 0 to 4 (19, 20). Level 0 is normal. Level 1 lesions are defined as irregular signals of the cartilage matrix, but the surface is intact. Level 2 lesions are superficial defects or cracks, accounting for <50% of the joint surface depth, while level 3 lesions represent ≥50% of the joint depth. Level 4 lesions are defined by full-thickness cartilage wear and subchondral bone exposure.

For bone marrow edema and ligaments (including anterior and posterior cruciate ligaments), the score of lesions ranged from 0 to 2. Level 0: normal. Level 1: the maximum diameter of the high signal seen on sagittal images is <10 mm (21). Level 2: the maximum diameter of the high signal is >10 mm.

For ligament lesions, a 3-point scale was used, divided into normal, partial tear, and complete tear. If the ligament displays a uniform low signal intensity and is continuous from the starting point to the ending point, it is regarded as normal. Partial tears are characterized by any one of indistinct ligament structure, local signal enhancement, visible edema and/or joint effusion around the ligament. Complete tears appear as discontinuities or disappearance of the ligaments.

Joint effusion was divided into presence or absence of effusion in axial and sagittal T2WI. Level 1: the maximum diameter of liquid in the suprapatellar bursa is <10 mm. Level 2: the maximum diameter of liquid in the suprapatellar bursa is >10 mm (21, 22). Measurement method: axial T2_DESSwe maximum distance before and after the maximum section of suprapatellar bursa effusion. 

### Statistical Analysis

Consistency between observers was tested using the intraclass correlation coefficient (ICC). SPSS 21.0 statistical software was used for statistical analysis, and *p*< 0.05 was considered statistically significant. Friedman’s test and Wilcoxon paired rank-sum test were used to compare quantitative indices of knee cartilage in the baseline, long-walking, and daily groups.

## Result

A total of 23 subjects (23 knees) were included in the study. There was good agreement in the measurement data between the observers (ICC = 0.813, 95% CI: 0.781-0.861). Comparison of injuries in different areas of the knee joint in three groups are as follows.

### Changes in the Level of the Meniscus, Cruciate Ligament, Cartilage Injury, Bone Marrow Edema, and Joint Effusion

Medial meniscus, anterior cruciate ligament, joint effusion, and bone marrow edema differed between the baseline and long-walking groups, but no difference was found in the daily group. Whereas lateral meniscus differed among the three groups, no difference in posterior cruciate ligament was observed among the three groups (**Tables 2**, **3**).

**Table 2:** Comparison of injurie level in different areas of the knee joint in three groups (n = 23).

meniscus	Baseline group		percentage	Long-walking group		percentage	Daily Group		percentage	X^2^ value	*p*-value
	medial	lateral		medial	lateral		medial	lateral			
0	15	12	58.7	9	5	30.43	18	19	80.44	15.854 (medial)	<0.001 (medial)
1	5	9	30.43	8	7	32.61	3	3	13.04		
2	3	1	8.70	4	8	26.09	1	1	4.35	28.182 (lateral)	<0.001 (lateral)
3	0	1	2.17	2	3	10.87	1	0	2.17		
4	0	0	0	0	0		0	0	0		
cartilage											
0	23		100	13		56.52	23		100	20	<0.001
1	0		0	3		13.04	0		0		
2	0		0	5		21.74	0		0		
3	0		0	1		4.35	0		0		
4	0		0	1		4.35	0		0		
Cruciate ligament	anterior	posterior		anterior	posterior		anterior	posterior			
0	23	23	100	19	23	91.3	23	23	100	8 (anterior)	0.018 (posterior)
1	0	0	0	4	0	8.7	0	0	0		
2	0	0	0	0	0	0	0	0	0	- (rear)	- (rear)
Bone marrow edema											
0	23		100	11		47.83	21		91.3	14	0.001
1	0		0	5		21.74	2		8.7		
2	0		0	7		30.43	0		0		
Joint effusion											
0	0		0	0		0	0		0	10	0.007
1	23		100	18		78.26	23		100		
2	0		0	5		21.74	0		0		

**Table 3:** Multiple comparisons of injuries in different areas of the knee joint (n = 23).

Region/injury	Baseline vs Long-walking group	Baseline vs Daily Group	Long-walking group vs Daily Group
Medial meniscus	0.005*	0.332	0.012*
Lateral meniscus	0.001*	0.007*	<0.001*
Cartilage	0.004*	1.000	0.004*
Anterior cruciate ligament	0.056	1.000	0.056
Posterior cruciate ligament	1.000	1.000	1.000
Joint effusion	0.025*	1.000	0.025*
Bone marrow edema	0.008*	0.328	0.008*

*Indicates statistical significance (p<0.05).

The degree of meniscus lesions significantly differed between the baseline and long-walking groups, and between the long-walking and daily groups. Nineteen menisci (41.30%) had level 1-3 lesions in the baseline group, 32 menisci (69.57%) had level 1-3 lesions in the long-walking group, and 9 menisci (19.56%) had level 1-3 lesions in the daily group.

**Figure 3:**
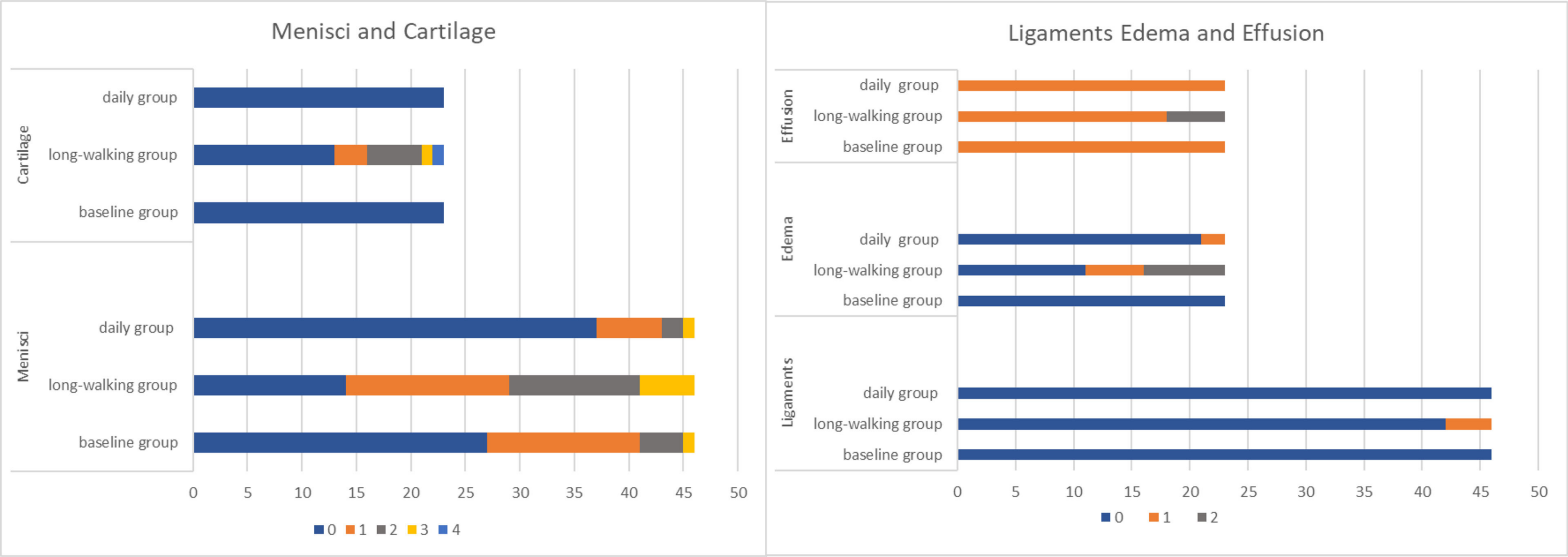
Changes in the level of the meniscus, cruciate ligament, cartilage injury, bone marrow edema, and joint effusion. (The horizontal axis represents the sample size, meniscus and ligament are twice as paired, and the different colors represent level).

No significant difference in anterior and posterior cruciate ligaments was found among the three groups. Only 4 (8.70%) subjects in the long-walking group had anterior cruciate ligament lesions, which were upgraded from level 0 to 1 (**Table 2** and **Figure 3**).

**Figure 4:**
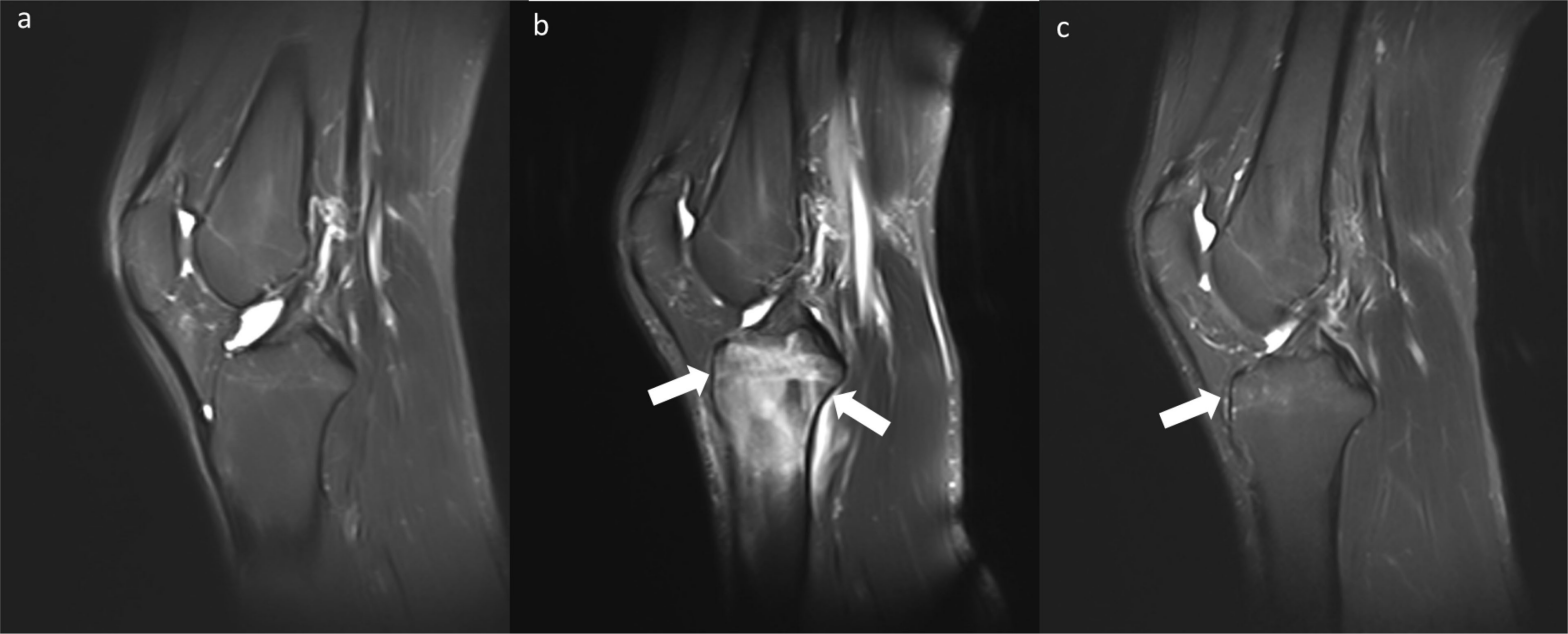
Changes in bone marrow edema (Level 0-2-1) in the baseline group **(A)**, long-walking group **(B)**, and daily group **(C)** on sagittal T2WI Dixon.

Bone marrow edema differed between the baseline and long-walking groups and between the long-walking and daily groups. No bone marrow edema lesions were observed in the baseline group (0%), and five cases (21.74%) of bone marrow edema lesions in the long-walking group upgraded from level 0 to 1, whereas seven cases (30.43%) upgraded to level 2. Only 2 subjects in the daily group had level 1 lesions (**Table 2**). One of the subjects had no bone marrow edema in the baseline group, but was upgraded to level 2 in the long-walking group and downgraded to level 1 in the daily group (**Figure 4**).

Furthermore, cartilage damage differed between the baseline and the long-walking groups, and between the long-walking and the daily groups. No cartilage damage (0%) was observed in the baseline and daily groups. In the long-walking group, three cases (13.04%) were upgraded from level 0 to level 1, five cases (21.74%) were upgraded to level 2, one case (4.35%) was upgraded to level 3, and one case (4.35%) was upgraded to level 4 lesions (**Table 2**).

Joint effusion differed between the baseline and the long-walking groups, and between the long-walking and the daily groups. Joint effusions were in level 1 in both the long-walking and daily groups. In the long-walking group, 18 cases (78.26%) were upgraded from level 0 to level 1 and five cases (21.74%) were upgraded to level 2 lesions (**Table 2**).

### Cartilage Volume

**Figure 5:**
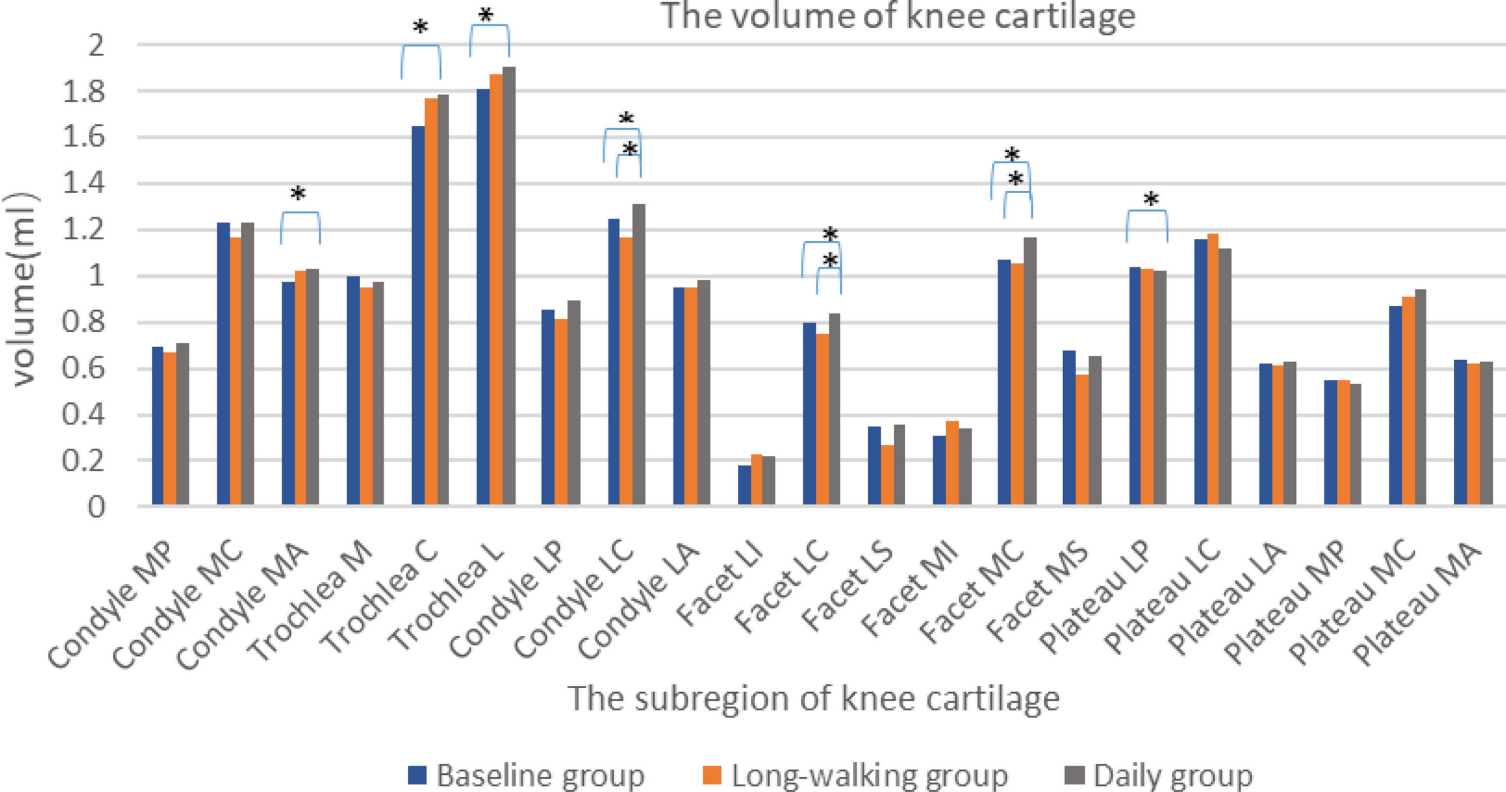
Histogram of volume changes in the sub-regions of the knee cartilage in the three groups. * Indicates statistical significance.

The volume of the sub-regions of knee articular cartilage, including Trochlea C, Trochlea L, Condyle MA, Condyle LC, Facet LC, Facet MC, and Plateau LP, differed between the baseline and daily groups, but no difference was found between the baseline and long-walking group. The volume of Trochlea C, Trochlea L, Condyle MA, Condyle LC, Facet MC, Facet LC increased after one year of daily training. However, Plateau LP volume decreased after one year of daily training. Besides, knee cartilage volume of other sub-regions did not change after one year of daily training in the long-walking group. Condyle LC, Facet LC, and Facet MC differed between the long-walking and daily groups, indicating that the volume of Condyle LC, Facet LC, and Facet MC increased after one year of daily training (**Table 4** and **Figure 5**).

**Table 4:** Volumes of the 21 sub-regions of the knee cartilage in the three groups.

Subregions	Baseline Group(ml)	Long-walking group (ml)	Daily group (ml)	X^2^ value	*p*-value
Condyle MP	0.690 (0.620, 0.790)	0.670 (0.610, 0.770)	0.710 (0.620, 0.790)	2.264	0.322
Condyle MC	1.230 (1.140, 1.370)	1.170 (1.010, 1.280)	1.230 (1.140, 1.440)	2.989	0.224
Condyle MA	0.970 (0.890, 1.050)	1.020 (0.930, 1.090)	1.030 (0.930, 1.120)	7.523	0.023*
Trochlea M	1.000 (0.880, 1.170)	0.950 (0.770, 1.100)	0.970 (0.850, 1.150)	3.303	0.192
Trochlea C	1.650 (1.440, 1.890)	1.770 (1.570, 2.170)	1.780 (1.600, 2.200)	8.330	0.016*
Trochlea L	1.810 (1.520, 2.060)	1.870 (1.680, 2.180)	1.900 (1.600, 2.220)	7.187	0.028*
Condyle LP	0.850 (0.750, 0.940)	0.810 (0.710, 0.900)	0.890 (0.780, 1.010)	5.826	0.054
Condyle LC	1.250 (1.060, 1.400)	1.170 (0.950, 1.420)	1.310 (1.180, 1.590)	11.822	0.003*
Condyle LA	0.950 (0.850, 1.060)	0.950 (0.850, 1.070)	0.980 (0.870, 1.110)	1.756	0.416
Facet LI	0.180 (0.160, 0.250)	0.230 (0.190, 0.280)	0.220 (0.180, 0.260)	0.161	0.923
Facet LC	0.800 (0.074, 0.850)	0.750 (0.062, 0.890)	0.840 (0.770, 0.950)	11.753	0.003*
Facet LS	0.350 (0.270, 0.460)	0.270 (0.240, 0.440)	0.360 (0.310, 0.470)	0.800	0.670
Facet MI	0.310 (0.270, 0.460)	0.370 (0.270, 0.460)	0.340 (0.270, 0.460)	0.159	0.924.
Facet MC	1.070 (0.920, 1.150)	1.050 (0.830, 1.140)	1.170 (1.020, 1.240)	13.130	0.001*
Facet MS	0.680 (0.400, 0.870)	0.570 (0.390, 0.790)	0.650 (0.500, 0.870)	1.253	0.535.
Plateau LP	1.040 (0.980, 1.220)	1.030 (0.940, 1.210)	1.020 (0.890, 1.140)	7.467	0.024*
Plateau LC	1.160 (1.030, 1.320)	1.180 (1.090, 1.250)	1.120 (1.000, 1.250)	0.843	0.666
Plateau LA	0.620 (1.030, 1.320)	0.610 (1.030, 1.320)	0.630 (1.030, 1.320)	2.000	0.368
Plateau MP	0.550 (0.520, 0.610)	0.550 (0.480, 0.620)	0.530 (0.480, 0.590)	2.956	0.228
Plateau MC	0.870 (0.840, 0.980)	0.910 (0.800, 0.970)	0.940 (0.800, 01.04)	0.629	0.730
Plateau MA	0.640 (0.560, 0.660)	0.620 (0.560, 0.670)	0.630 (0.560, 0.700)	0.161	0.923

The values are expressed as median (P25, P75) and compared using X^2^ and corresponding p values (n = 23). * Indicates statistical significance.

### Cartilage Thickness

**Figure 6:**
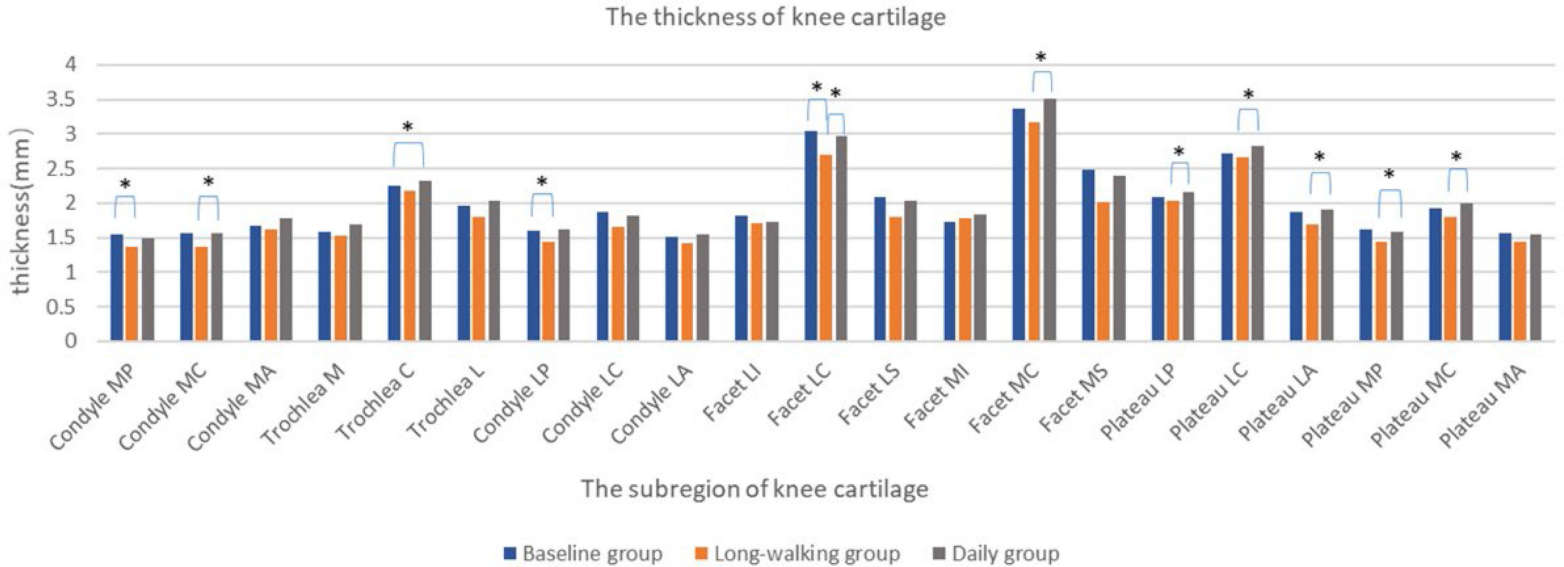
Changes in the thickness of each sub-area of the knee cartilage in the three groups. * Indicates statistical significance.

The thickness of knee articular cartilage in Condyle MC, Facet LC, Facet MC, Plateau LP, Plateau LC, Plateau LA, Plateau MP, Plateau MC significantly differed between the long-walking group and daily groups. Except for Trochlea C, which was increased, no differences were found in the other sub-regions between the baseline and the daily training groups. The thickness of Condyle MC, Facet LC, Facet MC, Plateau LP, Plateau LC, Plateau LA, Plateau MP, and Plateau MC was reduced after long-walking training. Condyle MP, Condyle LP, and Facet LC differed between the baseline and the long-walking groups, but no differences were found in the other sub-regions. The cartilage thickness of Condyle MP, Condyle LP, and Facet LC was significantly thinner in the long-walking group compared with the baseline group after training (**Table 5** and **Figure 6**).

**Table 5:** Thickness of the 21 sub-regions of the knee cartilage in the three groups.

Subregions	Baseline Group(mm)	Long-walking group(mm)	Daily group (mm)	X^2^ value	*p*-value
Condyle MP	1.540 (1.440, 1.680)	1.370 (1.140, 1.550)	1.500 (1.370, 1.660)	8.857	0.012*
Condyle MC	1.570 (1.440, 1.680)	1.370 (1.200, 1.630)	1.570 (1.420, 1.710)	18.067	<0.001*
Condyle MA	1.680 (1.580, 1.760)	1.620 (1.510, 1.730)	1.780 (1.670, 1.850)	4.242	0.120
Trochlea M	1.590 (1.490, 1.840)	1.520 (1.340, 1.750)	1.690 (1.540, 1.850)	3.231	0.199
Trochlea C	2.250 (1.890, 2.480)	2.180 (2.020, 2.590)	2.330 (2.120, 2.700)	7.187	0.028*
Trochlea L	1.960 (1.680, 2.160)	1.800 (1.670, 2.130)	2.030 (1.800, 2.210)	1.130	0.568
Condyle LP	1.600 (1.490, 1.780)	1.430 (1.390,1.690)	1.620 (1.510, 1.730)	7.977	0.019*
Condyle LC	1.870 (1.530, 2.120)	1.650 (1.470, 2.070)	1.810 (1.720, 2.200)	5.826	0.054
Condyle LA	1.510 (1.330, 1.640)	1.420 (1.340, 1.640)	1.540 (1.390, 1.730)	0.087	0.957
Facet LI	1.810 (1.570, 1.910)	1.710 (1.440, 2.120)	1.730 (1.550, 2.020)	0.261	0.878
Facet LC	3.050 (2.660, 3.320)	2.700 (2.400, 3.030)	2.970 (2.750, 3.160)	9.478	0.009*
Facet LS	2.080 (1.790, 2.360)	1.800 (1.510, 2.130)	2.030 (1.840, 2.220)	2.923	0.232
Facet MI	1.720 (1.540, 1.970)	1.780 (1.490, 1.860)	1.840 (1.690, 1.880)	0.087	0.957
Facet MC	3.370 (2.890, 3.680)	3.170 (2.780, 3.430)	3.510 (3.210, 3.790)	6.132	0.047*
Facet MS	2.480 (2.180, 2.910)	2.020 (1.760, 2.560)	2.400 (2.230, 2.900)	4.945	0.084.
Plateau LP	2.090 (2.040, 2.240)	2.030 (1.740, 2.170)	2.150 (2.080, 2.310)	10.876	0.004*
Plateau LC	2.710 (2.530, 2.800)	2.670 (2.430, 2.820)	2.820 (2.550, 2.970)	9.956	0.007*
Plateau LA	1.870 (1.680, 1.980)	1.690 (1.610, 1.91)	1.910 (1.820, 2.050)	6.879	0.032*
Plateau MP	1.620 (1.500, 1.700)	1.440 (1.270, 1.600)	1.590 (1.470, 1.710)	8.989	0.011*
Plateau MC	1.920 (1.830, 2.000)	1.800 (1.570, 1.990)	2.000 (1.880, 2.200)	14.957	0.001*
Plateau MA	1.560 (1.400, 1.650)	1.440 (1.330, 1.560)	1.540 (1.460, 1.710)	5.689	0.058

The values are expressed as median (P25, P75) and compared using X^2^ and corresponding p values (n = 23). * Indicates statistical significance.

### T2 Value

**Figure 7:**
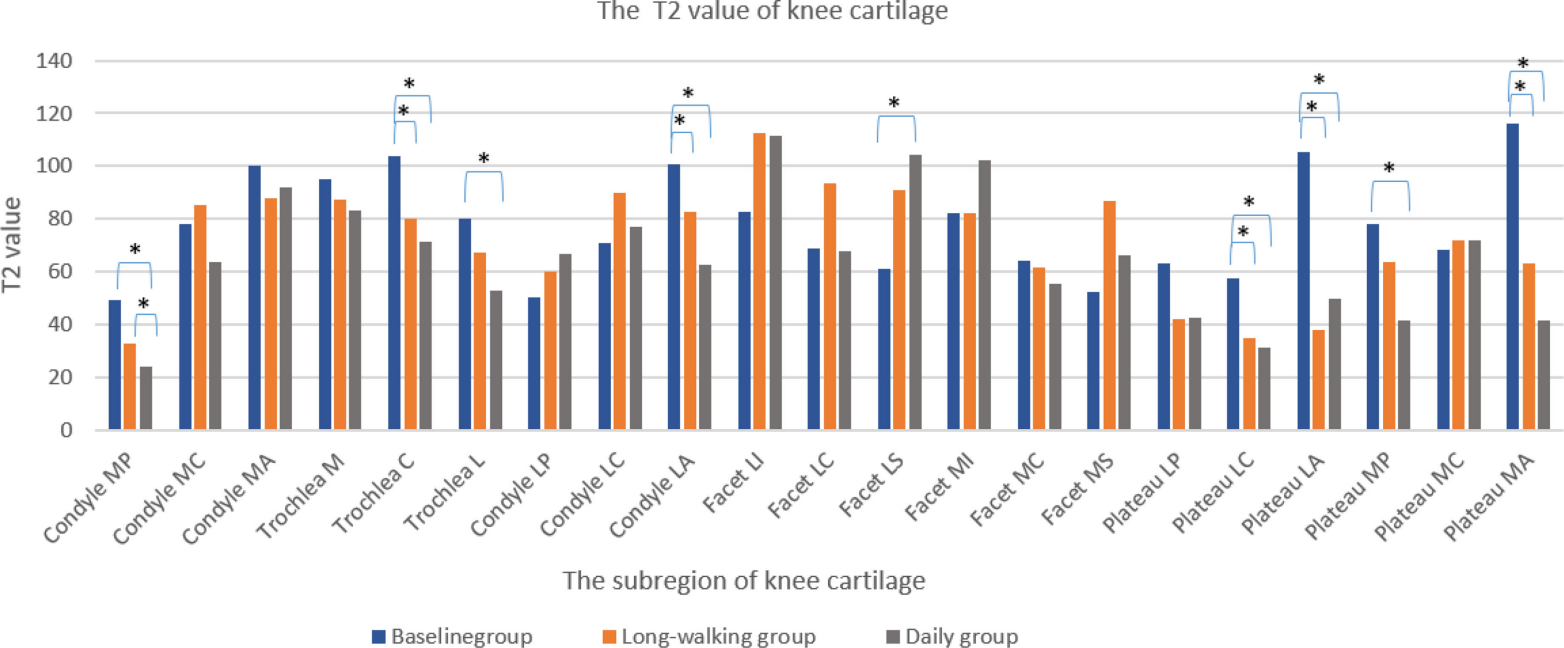
Changes in T2 values of knee cartilage in each sub-region in the three groups. * Indicates statistical significance.

T2 values in the sub-regions of knee cartilage, including Condyle MP, Trochlea C, Trochlea L, Condyle LA, Facet LS, Plateau LC, Plateau LA, Plateau MP, and Plateau MA, differed between the baseline and daily groups. T2 values of these subregions, except Facet LS, were lower in the daily training group than in the baseline group. However, except for Condyle MP, no difference was found between the daily and long-walking groups. This suggested that daily training and long walk have little effect on the T2 value. Moreover, Trochlea C, Condyle LA, Plateau LC, Plateau LA, and Plateau MA differed between the baseline and the long-walking groups, suggesting that the T2 values of these subregions decreased after training in the long-walking group (**Table 6** and **Figure 7**).

**Table 6:** T2 values of the 21 sub-regions of knee cartilage in the three groups.

Subregions	Baseline group	Long-walking group	Daily group	X^2^ value	*p*-value
Condyle MP	49.020 (35.760, 65.130)	32.770 (28.710, 37.310)	23.880 (21.050, 26.940)	19.826	<0.001*
Condyle MC	77.910 (69.390, 109.190)	85.250 (64.420, 112.360)	63.820 (57.680, 89.660)	3.217	0.200
Condyle MA	99.940 (86.610, 124.370)	87.830 (73.340, 109.130)	91.950 (78.560, 107.330)	1.826	0.401
Trochlea M	95.020 (81.800, 119.870)	87.370 (76.580, 110.760)	83.300 (70.950, 103.440)	0.783	0.676
Trochlea C	103.720 (74.210, 124.290)	79.890 (66.380, 90.470)	71.210 (57.330, 86.900)	11.565	0.003*
Trochlea L	80.350 (73.760, 96.360)	67.330 (57.540, 81.460)	52.900 (48.460, 62.930)	18.384	<0.001*
Condyle LP	50.410 (44.370, 77.360)	60.150 (51.690, 88.340)	66.510 (51.220, 116.150)	1.652	0.438
Condyle LC	70.980 (54.930, 101.840)	89.630 (72.530, 106.610)	77.050 (58.210, 96.440)	1.826	0.401
Condyle LA	100.830 (95.980, 116.260)	82.680 (72.430, 108.520)	62.420 (54.430, 79.670)	20.957	<0.001*
Facet LI	82.850 (62.310, 105.890)	112.320 (83.070, 132.480)	111.370 (74.70, 133.010)	3.217	0.200
Facet LC	68.730 (50.850, 76.840)	93.420 (54.750, 115.270)	67.700 (58.570, 91.140)	3.739	0.154
Facet LS	60.850 (1.040, 95.840)	90.980 (69.050, 134.020)	104.210 (71.260, 147.660)	7.304	0.026*
Facet MI	82.000 (72.640, 125.950)	81.970 (57.280, 109.480)	102.210 (81.080, 123.39)	3.130	0.209
Facet MC	64.310 (56.060, 77.880)	61.830 (41.520, 91.270)	55.210 (42.910, 75.350)	1.130	0.568
Facet MS	52.250 (44.370, 83.390)	86.740 (55.460, 122.120)	66.360 (56.300, 99.550)	5.478	0.065.
Plateau LP	63.020 (39.960, 107.890)	41.880 (35.230, 56.310)	42.480 (34.280, 51.080)	5.478	0.065
Plateau LC	57.260 (37.690, 86.260)	35.040 (29.790, 43.210)	31.280 (24.400, 39.820)	19.391	<0.001*
Plateau LA	105.550 (64.280, 139.260)	37.790 (30.580, 60.280)	49.670 (36.270, 71.280)	14.870	0.001*
Plateau MP	78.200 (49.290, 116.100)	63.490 (49.310, 112.050)	41.750 (29.860, 65.070)	8.435	0.015*
Plateau MC	68.220 (52.360, 96.730)	71.620 (46.150, 101.070)	71.620 (46.150, 101.070)	0.087	0.957
Plateau MA	116.340 (72.970, 136.200)	63.190 (42.290, 102.020)	41.500 (35.440, 76.820)	19.826	<0.001*

Values are expressed as is represented by the median (P25, P75), X^2^, and the corresponding p-value (n = 23). * Indicates statistical significance (p<0.05).

## Discussion

Sports injuries that accompany physical training restrict the effectiveness of training. Among them, knee injuries have the highest incidence and seriously affect daily activities (23). The present study recruited young male college freshmen. The pre-training image was used as a baseline for comparison. Eight-day long-walking training reflects knee joint injury caused by short-term, high-intensity exercise. One-year daily training can reflect the chronic damage to the knee joint caused by ordinary daily training. Previous studies have reported little knee joint injuries caused by long-walking training and daily training, and the follow-up time was about 1-3 months. However, reports on knee joint changes at ≥6 months are scarce (7, 24).

After long-walking training, the knee joint was subjected to high-load torsion, stretching, and compression forces. Laura et al. (24) reported similar results for knee joint meniscus, anterior cruciate ligament injury, bone marrow edema, and joint effusion to ours. Daily training intensity can be applied after recovery of the muscle strengthens, meniscus, anterior cruciate ligament, bone marrow edema, and joint effusion. The muscle strength in the baseline group was weak without regular systematic exercise. Insufficient hip muscle strength is an important factor leading to knee injuries. People with weak hip extensors have a more upright posture and over-rely on knee extensors during exercise, which leads to overuse of the knee joints. Hip muscle strengthening is beneficial to alleviate knee joint injury pain (25, 26). The 2017 guidelines of the ligament committee of the German Knee Society (DKG) proposed that external knee joint rotation is a high-risk action for the knee joint and anterior cruciate ligament injuries. It has been proven that jumping, running, flexibility stretching exercises, balance, and strength training can reduce the incidence of these injuries (27).

The thickness of cartilage is a two-dimensional parameter, while the volume is a three-dimensional parameter, and they have a certain correlation. Based on the 21 subregions of knee cartilage, combining the correlation and the physiological characteristics of the knee joint can be used for better sports injury analysis. Knee joint cartilage can produce elastic deformation and cushion vibration and its impact during exercise. Articular cartilage is composed of four areas: surface (tangential) area, intermediate (transition) area, deep (radial) area, and calcification area. The chondrocyte phenotype, cell shape, and extracellular matrix (ECM) structure vary in the different areas. The ECM is the main load-bearing component of the cartilage. Its major constituents are collagen (75% of dry weight) and proteoglycan (20-30% of dry weight), which vary in concentration with the depth of the talar joint surface. Surface collagen content is the highest, while proteoglycan content is the lowest. Proteoglycan polymer and interstitial fluid provide compressive elasticity for joints (28). Yoshioka et al. (29) found that the repair mechanism of cartilage damage in different directions of damage is different, and joint damage in parallel direction can be better repaired. Because the blood vessels of cartilage are only located in the surface, there is no blood vessel structure inside, which affects the transportation of repair cells, limiting the ability to repair the damage. Mature chondrocytes have limited proliferation potential and cannot produce enough ECM. Articular cartilage has functional adaptability to exercise. Exercise training can increase the thickness and compressibility of articular cartilage to ensure the compensation effect of the articular cartilage surface. Lu (30) found that knee cartilage volume was significantly reduced in young adults after 12 weeks of running and cycling, but had no significant change after non-impact swimming and low-impact power striding. Our data showed that knee joint cartilage volume increased after daily training. The main changes were the femoral stress surface, the patella, and the lateral area of the tibial plateau (Trochlea C, Trochlea L, Condyle MA, Condyle LC, Facet MC, and Facet LC). However, daily training had no significant effect on knee cartilage thickness. Our conclusion is similar to Eckstein (31). They examined triathletes, who had been training for at least 10 hours per week over the last three years and had been physically active also throughout childhood and adolescence. These were compared with individuals who had been never physically active (<1 hour sport per week throughout life), had no job involving any physical activity. They suggest that the mechanism to reduce stress may be by an increase in the bearing surface rather than by an increase in cartilage thickness in long-term training. Larger contact surface enhances pressurization. The review article also revealed a general principle of joint development, also in phylogenesis, large animals displaying much larger joint surfaces than man, but not necessarily thicker cartilage. In the long-walking training, the thickness of knee articular cartilage was compressed and thinned, and extensively involved the medial and lateral areas of the femur and tibial plateau (Condyle MP, Condyle LP, Condyle MC, Facet LC, Facet MC, Plateau LP, Plateau LC, Plateau LA, Plateau MP, and Plateau MC); however, no noticeable effect on the knee cartilage volume was observed. This finding suggested that only elastic deformation could be predominant in our short-term high-intensity training, and there is no difference between the medial and lateral. But, Jason (32) conducted a prospective cohort study based on the therapeutic exercise program of 13 patients with knee osteoarthritis. They completed a 6-week, twice per week outpatient training that included aerobic treadmill walking, lower extremity strengthening and flexibility exercises. Then complete 4 months of home training of the same intensity. The dynamic analysis showed that the therapeutic exercise method may increase the medial load of the knee joint and cause the reduction of medial femoral condyle cartilage. The loss of cartilage volume differed from our results due to different training methods and subjects.

T_2_ mapping utilizes the varying decay of transverse (T2) relaxation times of cartilage and provides information about the hydration status and collagen integrity of cartilage (33). The segmentation software segmented the T2 value of each subregion of the knee articular cartilage according to the T_2_ mapping. Long- walking and daily training reduced the T2 value of knee cartilage, and there was no difference in the reduction between the two training. Karanfil et al. (34) reached a similar conclusion that 30 minutes of running exercise can reduce the T2 value, but not the thickness of the knee cartilage. The two-phase medium hypothesis postulates that the movement pressure causes a reduction of the liquid composition to increase the buffering force, which leads to the decrease of the T2 value. The T2 value is associated with the content of free water in the cartilage and the arrangement direction of cartilage collagen fibers. If the collagen fibers in the cartilage disintegrate, irreversible cartilage damage will occur, leading to an increase in the T2 value (35, 36). Elevated T2 values predicted cartilage biochemical injury, however neither training increased.

Conventional MRI sequences can only evaluate cartilage morphology, while T_2_ mapping imaging can quantitatively analyze cartilage biochemical components and provide imaging evidence for the early diagnosis of cartilage damage. However, it is always time-consuming to manually draw the contours of the cartilage slice-by-slice of the 3D-DESSwe images in order to obtain quantitative parameters such as cartilage volume, thickness. Besides, the segmental results are operator dependent and is largely rely on the experiences of the processor. It is quite urgent and meaningful to use a tool for the automatic cartilage segmentation. It performs cartilage segmentation and evaluates biochemical maps. Specific knee, hip, and shoulder MR images can be processed using this software. The application allows users to process high-resolution 3D MR images with fat sat or water excitation plus biochemical maps (37, 38).

Our study has certain limitations. This study only involved the knee joint and did not study other structures, such as ankle joints and hip joints. Results reflect knee joint changes only for this sport condition and subjects. Unable to unify requirements for sports other than daily physical training subjects, which may affect our results. Subsequent studies should include females.

We demonstrated that long-walking training caused high-load torsion, tension, and compression of the knee joint. This damaged the knee meniscus and the anterior cruciate ligament and increased bone marrow edema and joint effusion. Daily training significantly increased knee cartilage volume, mainly in the stress surface of the femur, patella, and the lateral area of the tibial plateau, but knee cartilage thickness did not change. We come to conclusion, our results show that regular daily training does not cause high-level injury to the knee joint, but improve the knee joint function adaptability by increasing cartilage volume. Moreover, knee injury caused by short-term long walking can be reversible. This conclusion holds when similar to the experimental subjects as well as training method and intensity.

## Data Availability Statement

The original contributions presented in the study are included in the article/supplementary material. Further inquiries can be directed to the corresponding authors.

## Ethics Statement

The studies involving human participants were reviewed and approved by Ethics Committee of the First Affiliated Hospital of Army Medical University,PLA. Written informed consent to participate in this study was provided by the participants’ legal guardian/next of kin. Written informed consent was obtained from the individual(s), and minor(s)’ legal guardian/next of kin, for the publication of any potentially identifiable images or data included in this article.

## Author Contributions

LL and HL: Conceptualization, Methodology, Formal analysis, Investigation, Writing - Original Draft, Writing - Review & Editing, Visualization. ZZ and YZ: Investigation, Data Curation. XZ and ER: Software. JD: Project administration. YH, WC and XH: Validation, Writing - Review & Editing, Supervision, Funding acquisition. LL and HL contributed equally to this work, so they are listed as co-first authors. All authors contributed to the article and approved the submitted version.

## Funding

Research on the Prevention of Military Training Injuries of Army Officers and Soldiers (No.2019ZLX001) and Chongqing Key Clinical Specialty Construction Project (No.CQZDZK007)

## Conflict of Interest

XZ is an employee of Siemens Healthineers Ltd and ER is an employee of Siemens Healthcare GmbH.

The remaining authors declare that the research was conducted in the absence of any commercial or financial relationships that could be construed as a potential conflict of interest.

## Publisher’s Note

All claims expressed in this article are solely those of the authors and do not necessarily represent those of their affiliated organizations, or those of the publisher, the editors and the reviewers. Any product that may be evaluated in this article, or claim that may be made by its manufacturer, is not guaranteed or endorsed by the publisher.

## References

[1] Reynolds K Cosio-Lima L Bovill M Tharion W Williams J Hodges TA. A Comparison of Injuries, Limited-Duty Days, and Injury Risk Factors in Infantry, Artillery, Construction Engineers, and Special Forces Soldiers. Mil Med (2009) 174(7):702–8. doi: 10.7205/MILMED-D-02-2008 19685841

[2] Baker ML Epari DR Lorenzetti S Sayers M Boutellier U Taylor WR. Risk Factors for Knee Injury in Golf: A Systematic Review. Sports Med (Auckland NZ). (2017) 47(12):2621–39. doi: 10.1007/s40279-017-0780-5 PMC5684267 28884352

[3] McLean SG Lucey SM Rohrer S Brandon C. Knee Joint Anatomy Predicts High-Risk *In Vivo* Dynamic Landing Knee Biomechanics. Clin Biomech (Bristol Avon). (2010) 25(8):781–8. doi: 10.1016/j.clinbiomech.2010.06.002 20605063

[4] Miranda H Viikari-Juntura E Martikainen R Takala EP Riihimäki H. Physical Exercise and Musculoskeletal Pain Among Forest Industry Workers. Scand J Med Sci Sports. (2001) 11(4):239–46. doi: 10.1034/j 11476430

[5] Shin D Youn K Lee E Lee M Chung H Kim D. Risk Factors for Lesions of the Knee Menisci Among Workers in South Korea’s National Parks. Ann Occup Environ Med (2016) 28:56. doi: 10.1186/s40557-016-0143-y 27766160 PMC5057211

[6] Leiter JRS MacDonald L McRae S Davidson M MacDonald PB. Intrinsic Stresses on Bone and Cartilage in the Normal and Anterior Cruciate Ligament-Reconstructed Knee Before and After a Half Marathon: A Magnetic Resonance Imaging Analysis. Clin J Sport Med (2012) 22(5):439–42. doi: 10.1097/JSM.0b013e31825d0d4a 22722732

[7] Qiu L Perez J Emerson C Barrera CM Zhong J Nham F. Biochemical Changes in Knee Articular Cartilage of Novice Half-Marathon Runners. J Int Med Res (2019) 47(11):5671–9. doi: 10.1177/0300060519874140 PMC6862918 31566042

[8] Schutz U Ehrhardt M God S Billich C Beer M Trattnig S. A Mobile MRI Field Study of the Biochemical Cartilage Reaction of the Knee Joint During a 4,486 Km Transcontinental Multistage Ultra-Marathon Using T2* Mapping. Sci Rep (2020) 10(1):8157. doi: 10.1038/s41598-020-64994-2 32424133 PMC7235258

[9] Zhang Y Shu D Yao W Ding J Chen L Lin X. MRI Study of Changes in Knee Bone Marrow Edema-Like Signal in Asymptomatic Amateur Marathon Runners Before and After Half-Marathon Running. Clin Imaging (2021) 80:150–7. doi: 10.1016/j.clinimag.2021.05.005 34332463

[10] Glaser C Faber S Eckstein F Fischer H Springer V Heudorfer L. Optimization and Validation of a Rapid High-Resolution T1-W 3D FLASH Water Excitation MRI Sequence for the Quantitative Assessment of Articular Cartilage Volume and Thickness. Magn Reson Imaging (2001) 19(2):177–85. doi: 10.1016/S0730-725X(01)00292-2 11358655

[11] Graichen H Jakob J von Eisenhart-Rothe R Englmeier KH Reiser M Eckstein F. Validation of Cartilage Volume and Thickness Measurements in the Human Shoulder With Quantitative Magnetic Resonance Imaging. Osteoarthritis Cartilage (2003) 11(7):475–82. doi: 10.1016/S1063-4584(03)00077-3 12814610

[12] Wang Z Ai S Tian F Liow MHL Wang S Zhao J. Higher Body Mass Index Is Associated With Biochemical Changes in Knee Articular Cartilage After Marathon Running: A Quantitative T2-Relaxation MRI Study. Orthop J Sports Med (2020) 8(8):2325967120943874. doi: 10.1177/2325967120943874 32851106 PMC7427140

[13] Hou W Zhao J He R Li J Ou Y Du M. Quantitative Measurement of Cartilage Volume With Automatic Cartilage Segmentation in Knee Osteoarthritis. Clin Rheumatol (2021) 40(5):1997–2006. doi: 10.1007/s10067-020-05388-7 33026551

[14] Qin Y Zhang J Li P Wang Y. 3D Double-Echo Steady-State With Water Excitation MR Imaging of the Intraparotid Facial Nerve at 1.5T: A Pilot Study. AJNR Am J Neuroradiol (2011) 32(7):1167–72. doi: 10.3174/ajnr.A2480 PMC7966035 21566007

[15] Surowiec RK Lucas EP Fitzcharles EK Petre BM Dornan GJ Giphart JE. T2 Values of Articular Cartilage in Clinically Relevant Subregions of the Asymptomatic Knee. Knee Surgery Sports TraumatoL Arthroscopy (2014) 22(6):1404–14. doi: 10.1007/s00167-013-2779-2 24271329

[16] Fripp J Crozier S Warfield SK Ourselin S. Automatic Segmentation and Quantitative Analysis of the Articular Cartilages From Magnetic Resonance Images of the Knee. IEEE Trans Med Imaging (2010) 29(1):55–64. doi: 10.1109/TMI.2009.2024743 19520633 PMC3717377

[17] Newe A. Towards an Easier Creation of Three-Dimensional Data for Embedding Into Scholarly 3D PDF (Portable Document Format) Files. PeerJ (2015) 3:e794. doi: 10.7717/peerj.794 25780759 PMC4358654

[18] Kocabey Y Tetik O Isbell WM Atay OA Johnson DL. The Value of Clinical Examination Versus Magnetic Resonance Imaging in the Diagnosis of Meniscal Tears and Anterior Cruciate Ligament Rupture. Arthroscopy (2004) 20(7):696–700. doi: 10.1016/S0749-8063(04)00593-6 15346110

[19] Outerbridge R. The Etiology of Chondromalacia Patellae. J Bone Joint Surg [Br] (1961) 43-B(4):752–7. doi: 10.1302/0301-620X.43B4.752 14038135

[20] Link TM Vieth V Stehling C Lotter A Beer A Newitt D. High-Resolution MRI vs Multislice Spiral CT: Which Technique Depicts the Trabecular Bone Structure Best? Eur Radiol (2003) 13(4):663–71. doi: 10.1007/s00330-002-1695-5 12664101

[21] Schueller-Weidekamm C Schueller G Uffmann M Bader TR. Does Marathon Running Cause Acute Lesions of the Knee? Evaluation With Magnetic Resonance Imaging. Eur Radiol (2006) 16(10):2179–85. doi: 10.1007/s00330-005-0132-y 16528558

[22] Schueller-Weidekamm C Schueller G Uffmann M Bader T. Incidence of Chronic Knee Lesions in Long-Distance Runners Based on Training Level: Findings at MRI. Eur J Radiol (2006) 58(2):286–93. doi: 10.1016/j.ejrad.2005.11.010 16368218

[23] Jones BH Knapik JJ. Physical Training and Exercise-Related Injuries. Surveillance, Research and Injury Prevention in Military Populations. Sports Med (Auckland NZ). (1999) 27(2):111–25. doi: 10.2165/00007256-199927020-00004 10091275

[24] Horga LM Henckel J Fotiadou A Hirschmann AC Di Laura A Torlasco C. Is the Immediate Effect of Marathon Running on Novice Runners’ Knee Joints Sustained Within 6 Months After the Run? A Follow-Up 3.0 T MRI Study. Skeletal Radiol (2020) 49(8):1221–9. doi: 10.1007/s00256-020-03391-2 PMC7300102 32065245

[25] Teng HL Powers CM. Hip-Extensor Strength, Trunk Posture, and Use of the Knee-Extensor Muscles During Running. J Athl Train (2016) 51(7):519–24. doi: 10.4085/1062-6050-51.8.05 PMC5317187 27513169

[26] Marn-Vukadinovic D Bizovicar N Majdic N Vidmar G. Pain and Outcome Prediction in Muscle Strength Rehabilitation After Knee Injury in Recreational Athletes. Int J Rehabil Res (2019) 42(2):168–73. doi: 10.1097/MRR.0000000000000342 31034452

[27] Mehl J Diermeier T Herbst E Imhoff AB Stoffels T Zantop T. Evidence-Based Concepts for Prevention of Knee and ACL Injuries. 2017 Guidelines of the Ligament Committee of the German Knee Society (DKG). Arch Orthop Trauma Surg (2018) 138(1):51–61. doi: 10.1007/s00402-017-2809-5 28983841

[28] Carballo CB Nakagawa Y Sekiya I Rodeo SA. Basic Science of Articular Cartilage. Clin Sports Med (2017) 36(3):413–25. doi: 10.1016/j.csm.2017.02.001 28577703

[29] Yoshioka M Kubo T Coutts RD Hirasawa Y. Differences in the Repair Process of Longitudinal and Transverse Injuries of Cartilage in the Rat Knee. Osteoarthritis Cartilage (1998) 6(1):66–75. doi: 10.1053/joca.1997.0093 9616440

[30] Lu L Wang Y. Effects of Exercises on Knee Cartilage Volume in Young Healthy Adults: A Randomized Controlled Trial. Chin Med J (2014) 127(12):2316–21. doi: 10.3760/cma.j.issn.0366-6999.20131991 24931249

[31] Eckstein F Reiser M Englmeier KH Putz R. *In Vivo* Morphometry and Functional Analysis of Human Articular Cartilage With Quantitative Magnetic Resonance Imaging–From Image to Data, From Data to Theory. Anat Embryol (Berl) (2001) 203(3):147–73. doi: 10.1007/s004290000154 11303902

[32] Woollard JD Gil AB Sparto P Kwoh CK Piva SR Farrokhi S. Change in Knee Cartilage Volume in Individuals Completing a Therapeutic Exercise Program for Knee Osteoarthritis. J Orthop Sports Phys Ther (2011) 41(10):708–22. doi: 10.2519/jospt.2011.3633 PMC3383656 21891881

[33] Apprich SR Schreiner MM Szomolanyi P Welsch GH Koller UK Weber M. Potential Predictive Value of Axial T2 Mapping at 3 Tesla MRI in Patients With Untreated Patellar Cartilage Defects Over a Mean Follow-Up of Four Years. Osteoarthritis Cartilage (2020) 28(2):215–22. doi: 10.1016/j.joca.2019.10.009 31678665

[34] Karanfil Y Babayeva N Donmez G Diren HB Eryilmaz M Doral MN. Thirty Minutes of Running Exercise Decreases T2 Signal Intensity But Not Thickness of the Knee Joint Cartilage: A 3.0-T Magnetic Resonance Imaging Study. Cartilage (2019) 10(4):444–50. doi: 10.1177/1947603518770246 PMC6755866 29676169

[35] Juras V Schreiner M Laurent D Zbyn S Mlynarik V Szomolanyi P. The Comparison of the Performance of 3T and 7T T2 Mapping for Untreated Low-Grade Cartilage Lesions. Magn Reson Imaging (2019) 55:86–92. doi: 10.1016/j.mri.2018.09.021 30244140 PMC6420148

[36] Collins AT Hatcher CC Kim SY Ziemian SN Spritzer CE Guilak F. Selective Enzymatic Digestion of Proteoglycans and Collagens Alters Cartilage T1rho and T2 Relaxation Times. Ann BioMed Eng (2019) 47(1):190–201. doi: 10.1007/s10439-018-02143-7 30288634 PMC6481190

[37] Zhang P Zhang RX Chen XS Zhou XY Raithel E Cui JL. Clinical Validation of the Use of Prototype Software for Automatic Cartilage Segmentation to Quantify Knee Cartilage in Volunteers. BMC Musculoskelet Disord (2022) 23(1):19. doi: 10.1186/s12891-021-04973-4 34980107 PMC8725480

[38] Zhang P Yu B Zhang R Chen X Shao S Zeng Y. Longitudinal Study of the Morphological and T2* Changes of Knee Cartilages of Marathon Runners Using Prototype Software for Automatic Cartilage Segmentation. Br J Radiol (2021) 94(1119):20200833. doi: 10.1259/bjr.20200833 33544636 PMC8011266

